# Investigation of the Structural Behavior of Reinforced Concrete Beams at Elevated Temperatures

**DOI:** 10.1021/acsomega.3c09403

**Published:** 2024-02-14

**Authors:** Mahmud Yağan, Fatih Mehmet Özkal, Muhammed Orhan Öztürk, Mehmet Polat

**Affiliations:** Department of Civil Engineering, Atatürk University, 25240 Erzurum, Turkey

## Abstract

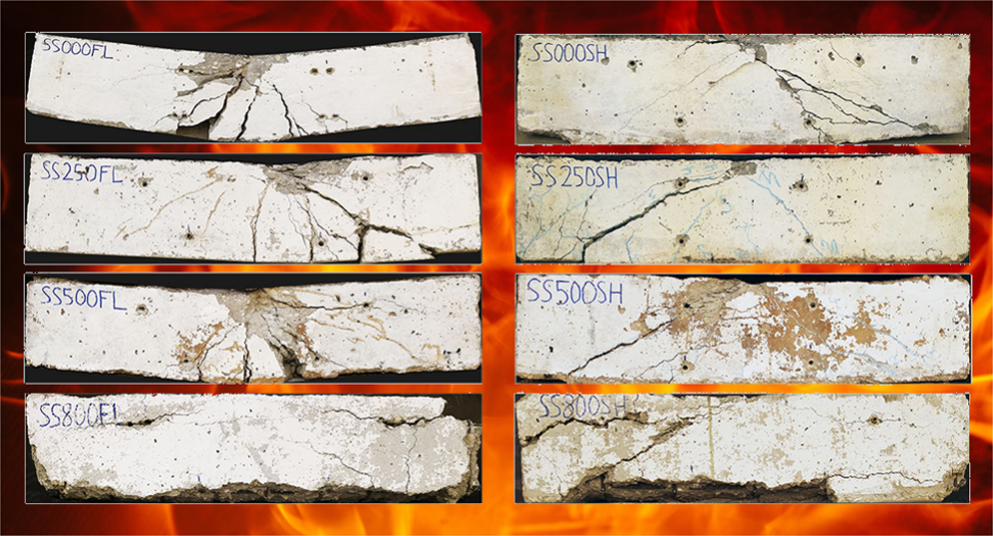

Reinforced concrete structures encounter a range of detrimental external factors over their operational lifespan. One of them is the elevated temperature effect due to fires. Conversely, due to the influence of global warming, temperatures are on the rise worldwide, leading to an increase in fire incidents. Owing to the increasing rates of construction and fire incidents, it becomes imperative to investigate the durability of reinforced concrete members when exposed to high temperatures. This experimental study aims to assess the structural behavior of reinforced concrete beams following exposure to elevated temperatures. To accomplish this goal, concrete cube specimens, steel rebars, pull-out specimens, and reinforced concrete beams were exposed to elevated temperatures of up to 800 °C and then allowed to cool in air. Following this, all specimens were subjected to testing in accordance with the relevant codes and standards. Test results were analyzed through comparison. In a comprehensive examination of the results, it is evident that the concrete compressive strength experiences an approximately 55% reduction at 600 °C. Meanwhile, there is no notable decrease in the yield strength of the steel at this temperature. However, at 800 °C, steel yield strength decreases by nearly 30%, while the compressive strength of the concrete decreases by a significant 82%. This indicates a substantial reduction in the load-bearing capacity of the beam specimens due to concrete deterioration and the subsequent decline in the bonding performance between concrete and steel rebars.

## 1 Introduction

Reinforced concrete structures face a multitude of adverse external incidents over their service life. Among these factors, elevated temperatures resulting from fires are a significant concern. Considering other respects, global warming is contributing to a steady rise in temperatures worldwide, leading to a surge in fire incidents. Given the escalating rates of construction and fire occurrences globally, it becomes imperative to scrutinize the resilience of reinforced concrete members when exposed to elevated temperatures caused by fires. This study focuses on assessing the impact of elevated temperatures on reinforced concrete beams and offers a comprehensive review of the relevant literature.

To gain a comprehensive understanding of the fire resistance of reinforced concrete structures, it becomes crucial to comprehend how elevated temperatures affect the bond strength between concrete and reinforcement. Morley and Royles,^1^ as one of the pioneering studies that took into account the elevated temperature effects on construction materials, examined the strength deterioration of steel and concrete under elevated temperatures. Past research on bond strength at ambient and elevated temperatures was evaluated and recommendations on structural performance were presented. Rostásy et al.^2^ investigated the change in the porous structure of concrete under elevated temperatures (up to 900 °C) using the mercury porosimetry method and revealed that the pore volume of the concrete expands as the temperature increases. El-Hawary et al.^3^ performed an experimental investigation aiming to elucidate the bonding characteristics at the interface between reinforcement and concrete when subjected to elevated temperatures. Furthermore, they explored how variables such as heating duration, cooling techniques, and temperature levels influence the bond performance. Steel reinforcement in reinforced concrete elements is protected from environmental factors by a concrete cover. According to Akman,^4^ when the behavior of steel under the influence of elevated temperature is investigated, it is apparent that although the tensile strength of steel increases due to the diffusion of nitrogen atoms to the grain boundaries where dislocations are intense at 200 °C, the tensile and yield limits will decrease at 300 °C, the tensile strength at 600 °C will fall below the safety zone, and plastic deformation will occur at 600–1200 °C during a fire. In structural members exposed to elevated temperatures, decreases are also observed in the modulus of elasticity of steel under stress, 15% at 400 °C and 40% at 600 °C. This decrease will cause excessive elongation of the steel as a result of the initiation of thermal expansion and plastic deformations. Considering the necessity of protection from the elevated temperature effect, it is seen that concrete also protects steel reinforcement from the elevated temperature effect. In this case, it becomes important for the concrete to provide the covering thickness (placer) and the necessary thermal insulation.^4^ If the maximum temperature under the influence of an elevated temperature is less than 450 °C in cold-treated steels and less than 600 °C in hot-rolled steels, the yield strength is regained after cooling.^5^

According to studies conducted by Xiao and König,^6^ when the temperature increases from room temperature to 400 °C, steel strength merely increases alongside ductility reduction. At temperatures above this, the strength of steel decreases regularly, and at 700 °C, only 20% of the initial strength remains. The modulus of elasticity of steel also decreases regularly with a temperature increment. Since the thermal expansion of concrete at elevated temperatures is much lower than the expansion of the reinforcing steel, the compression of the concrete around the reinforcement increases the friction between the concrete and the reinforcement. On the other hand, the tensile strength of concrete decreases. For this reason, the adherence between the concrete and the reinforcement, which has been changed by elevated temperature effects, affects the crack, deformation, and load-carrying capacity of the reinforced concrete elements exposed to fire. It can be said that the bond of concrete-reinforcement changed very distinctly after the effects of an elevated temperature. The amount of bond deterioration is greater than the compressive strength decrease of concrete.

Bingöl and Gül^7^ conducted a study to examine the bond strength between steel reinforcement and concrete after exposure to elevated temperature. With an increasing temperature level, the losses in bond stress and concrete compressive strength were determined. It has been stated that higher losses in water-cooled samples may be due to thermal shock caused by sudden temperature changes. Decarburization generally means a decrease in the carbon content on the material surface. Steels provide the desired micro structure and mechanical properties by heat treatment. These processes are usually in the austenite field and are between 800 and 1200 °C depending on the chemical composition. The presence of oxygen in the furnace atmosphere is inevitable. In addition, the carbon content in the atmosphere may be lower than the carbon content in the heat-treated material. For this reason, carbon tends to separate from the steel surface and this is called decarburization.^8^

Xiao et al.^9^ undertook a research project to empirically explore the shear transfer behavior of high-strength concrete following exposure to elevated temperatures along a premade crack. The study primarily focused on assessing two key variables: the compressive strength of the concrete and the magnitude of the elevated temperature. Pressure tests with crack-free specimens were performed to examine the shear strength of concrete regarding fracture formation and deformation after elevated temperatures. As a result, it has been observed that the final shear strength decreases at temperatures above 200 °C in high-strength concrete and the corresponding deterioration form (fracture slip and fracture wideness) increases with increasing temperature. Panedpojaman et al.^10^ conducted fire tests to investigate the bond properties of reinforced concrete beams with a share of rust of different values at elevated temperatures. As a result of the experiments conducted after elevated temperature, it was asserted that the fracture types of reinforced concrete beams are shear fracture, flexural fracture, and shear fracture with tensile splitting cracks. In addition, it has been stated that samples with a rust share of 25 mm receive more damage due to an insufficient rust share and collapse earlier. Mwamlamba et al.^11^ showed that the flexural strength loss of reinforced concrete beams increases up to 20–25% after being exposed to a temperature of 250 °C. Similarly, Fathi et al.^12^ reported that as the temperature increases, the maximum yield strength and flexural strength of reinforced concrete beams decrease. Kadhum et al.^13^ studied the fire effect on drying shrinkage, compressive strength, and load-deflection behavior of reinforced concrete beams.

Özkal et al.^14^ studied the mechanical and bonding characteristics of GFRP and steel rebars exposed to elevated temperature effects within a correlated comparison. After exposure to elevated temperature effects in the range of 23–800 °C, pull-out tests and axial tensile tests were performed. As a result of the study, despite the use of limestone-based aggregate and silica-based cement in concrete, concrete samples were able to maintain 45% of the initial compressive strength at 600 °C and 18% at 800 °C. When the axial tensile test results of GFRP and steel rebars exposed to elevated temperature impacts without any protection were examined, it was found that there was only about 30% loss at 800 °C, although there was no change in the steel yield strength up to 600 °C. In addition, as a result of the study, a mathematical model was proposed and experimental results were compared with this model. As a result of the comparison, it was stated that the reinforcement showed a consistent attitude in terms of the bond strength. It was concluded that this empirical modeling has the capacity to estimate the bond strength of rebar at elevated temperatures and can minimize the need for any extensive experimental work.

A study was conducted by Xing et al.^15^ to examine the shear behavior of reinforced concrete beams after exposure to elevated temperatures. Three sides of each beam were exposed to ISO 834-1 standard fire for 2 h, and then shear loading was applied on the beams. Test results showed that shear-bearing capacity and shear stiffness decrease with the temperature and the final deformations of the beams increase. They also concluded that the decrease in the flexural bearing capacity of the beams was greater than the decrease in the shear-bearing capacity when the pressure zone was exposed to elevated temperatures.

Hassan et al.^16^ conducted a study on the effect of elevated temperature levels on concrete and the effect of different reinforcement techniques on restoring the capacity of reinforced concrete beams. Test specimens were subjected to temperatures of 400 and 600 °C for 60 and 120 min to evaluate the beam behavior under fire conditions using different types of concrete. It was concluded that the duration of exposure to elevated temperature and different types of concrete are effective in reinforced concrete members, and there is a significant loss of strength, especially in the specimens produced with normal concrete subjected to 600 °C for 2 h.

Ahmad et al.^17^ tested concrete specimens to examine the effect of elevated temperatures on the shear capacity. The specimens produced with 40 MPa concrete were subjected to temperatures up to 350, 550, and 750 °C, then naturally cooled to room temperature, and subjected to the shear tests. As a result of the investigation, it was revealed that the shear capacity of concrete exposed to elevated temperatures decreased by 18.85% at 350 °C, 29.6% at 550 °C, and 52.74% at 750 °C.

Aliş et al.^18^ performed finite element analyses in order to attain the flexural behavior of reinforced concrete beams exposed to elevated temperatures. The study was based on the ISO-834 fire curve, considering various fire durations and temperature levels. In addition, an algorithm has been developed by combining nonlinear finite element analysis and thermal analysis. It was mentioned that the numerical model created is compatible with experimental studies, and this model can be used to design and optimize fire protection systems in order to obtain cost-effective solutions.

## 2 Material Characteristics

In this section, the characteristics of the reinforced concrete materials are presented under three headings: concrete compressive strength, steel tensile strength, and concrete-steel pull-out tests. Material test results in this section could also be found in the studies of Polat et al.^19^ and Özkal et al.^14^

### 2.1 Concrete Compressive Strength Tests

Concrete has an important role within the research on elevated temperature effects on reinforced concrete members. It can be said that if the temperature effect increases, the mechanical properties of the reinforcing rebars in concrete are indirectly affected. Test specimens were produced by the ready-mixed concrete with the largest grain diameter of 15 mm, the 28-day compressive strength of 45 MPa and the slump value of 20 cm. In order to acquire the concrete strength, three cube samples with dimensions of 15 × 15 × 15 cm were taken for each test group at different temperatures. These samples were kept in curing pools under the same conditions as reinforced concrete beams, placed in the electric furnace, exposed to elevated temperatures for 60 min, and then left to cool at room temperature.

**Figure 1:**
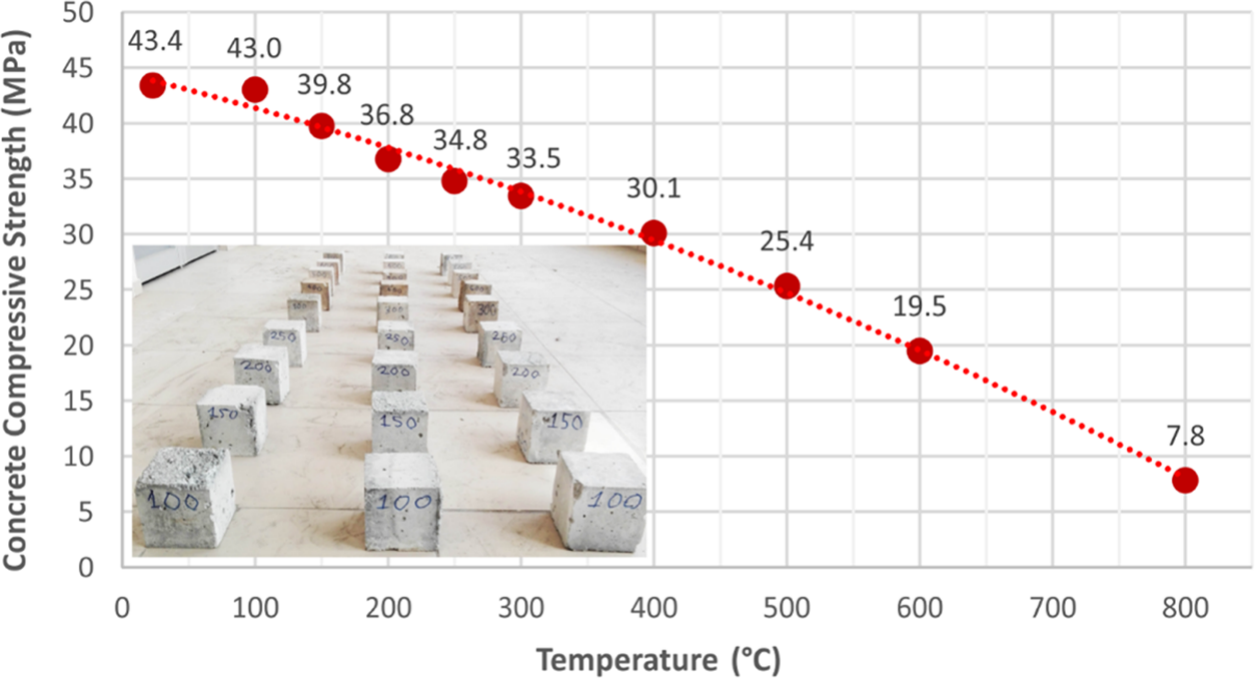
Compressive strength of concrete exposed to elevated temperatures.

According to the test results conducted with a loading speed of 0.25 MPa/s in accordance with ASTM C39/C39M-17b,^20^ the decreasing curve of concrete compressive strength at elevated temperature levels is shown in Figure 1. Although cylindrical concrete samples are recommended for compressive strength testing in ASTM C39/C39M-17b,^20^ the authors preferred to use cubic concrete samples to ensure a better fit with pull-out and reinforced concrete beam samples.

As can be seen from Figure 1, there was no serious decrease in concrete compressive strength up to 150 °C. There was a 15% decrease in concrete compressive strength at 200 °C compared to the control specimens (23 °C), while there was a 20% decrease at 250 °C, a 22% decrease at 300 °C, and a 30% decrease at 400 °C. When higher elevated temperatures were reached, a decrease of 41% was observed at 500 °C and 55% at 600 °C. At 800 °C, concrete has almost lost integrity, and there has been an 82% decrease in concrete compressive strength.

Since concrete has a complex structure and its components (aggregate and cement) contain silica and limestone, the loss of strength is expected to depend on various parameters. Quartz, particularly in silica-based coarse and fine aggregates, undergoes a polymorphic shift when exposed to a temperature of 570 °C, transitioning from α quartz to β quartz. This alteration results in an expansion of volume and can lead to concrete damage.^21,22^ Furthermore, in calcareous and dolomitic aggregates, carbonates transform into CaO or MgO within the range 800–900 °C. With rising temperatures, limestone or dolomite expands, and the decomposition of CO_2_ and the formation of CaO or MgO trigger a contraction. These shifts in volume also contribute to concrete damage.^22,23^ According to the findings of this investigation, concrete maintains 45% of its compressive strength at 600 °C, but this strength diminishes to a mere 18% at 800 °C. These results underscore the superior material performance of concrete at high temperatures compared to earlier publications.

In Eurocode 2^24^ (Part 1–2), it is assumed that concrete compressive strength can decrease to 60% at 500 °C, 45% at 600 °C, and 15% at 800 °C, and this claim is consistent with the results of this research (500 °C: 58%; 600 °C: 45%; 800 °C: 18%).

### 2.2 Steel Tensile Strength Tests

**Figure 2:**
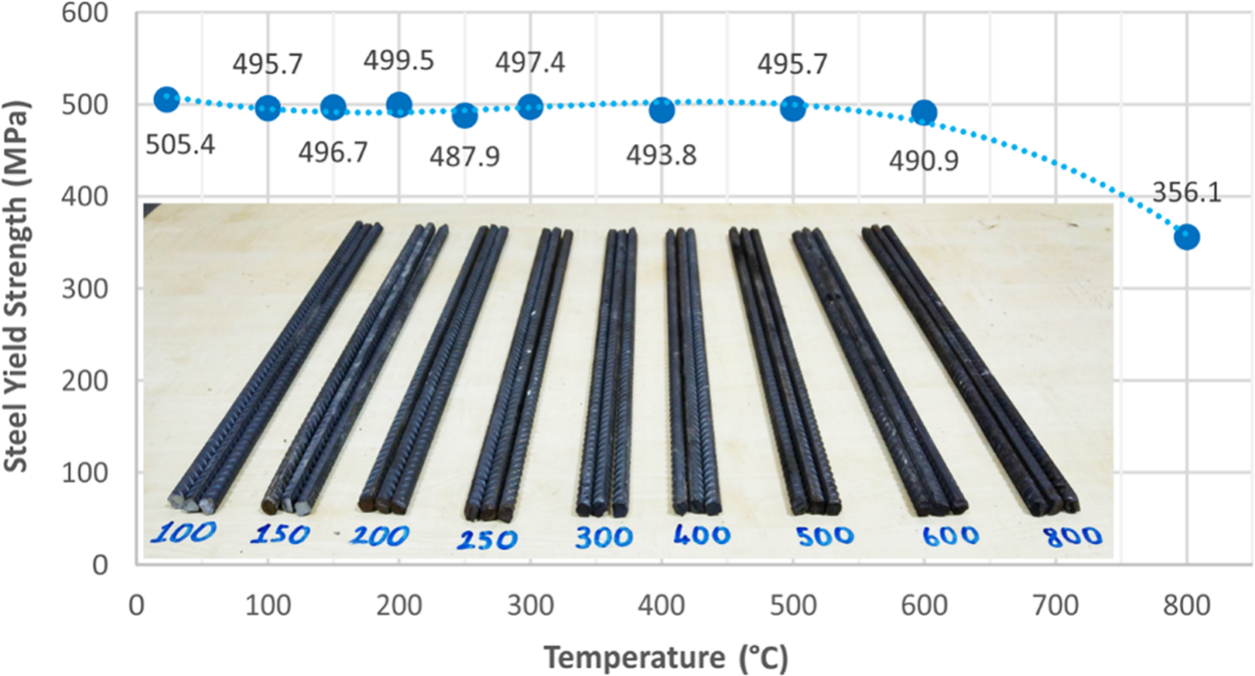
Yield strength of steel exposed to elevated temperatures.

Hot-rolled and cold-formed steels exhibit different behavior against exposure of elevated temperatures.^5^ S420 class hot-rolled steel was used in this study. Since the yield strength of the hot-rolled steel is largely preserved after cooling, fire plaster was not required for the axial tensile samples. To assess the mechanical properties of steel reinforcement subjected to high temperatures, all samples underwent a gradual exposure to temperatures ranging from 100 to 800 °C for 60 min. Following this exposure, the samples were allowed to cool to room temperature, with the exception of control specimens. After that, in accordance with the recommendations of ASTM A370-17,^25^ axial tensile tests of three rebars were performed with a loading speed of 2 mm/min in each temperature group. Yield strength values of steel rebars (db = 12 mm) for each temperature group are shown in Figure 2.

Regarding previous studies, it is expected that the elasticity modulus and yield strength values of steel tend to decrease at elevated temperatures. Milke^26^ noted that conventional structural steel undergoes crystallization at approximately 650 °C. According to Lie^27^ and SFPE,^28^ it has been documented that steel typically maintains 50% of its tensile strength and rigidity under environmental conditions at a similar temperature of 593 °C (1000 °F) as concrete. The reduction in these properties increases to 80% at 700 °C and approaches nearly 100% at 1200 °C.^14,29,30^ If the exposed temperature in hot-rolled steels is less than 600 °C, the yield strength is regained after cooling.^5^

The test results reveal that there is no noteworthy alteration in the yield strength of steel bars up to 600 °C. However, a substantial decrease of nearly 30% in yield strength becomes apparent at 800 °C. Surprisingly, this study uncovered tensile strength outcomes for steel bars that deviate from conventional expectations and earlier research findings. Hence, it can be inferred that the material properties of steel have progressed over time, rendering it suitable for use even at temperatures as high as 800 °C, as suggested by Yağan.^31^

### 2.3 Concrete-Steel Pull-Out Tests

**Figure 3:**
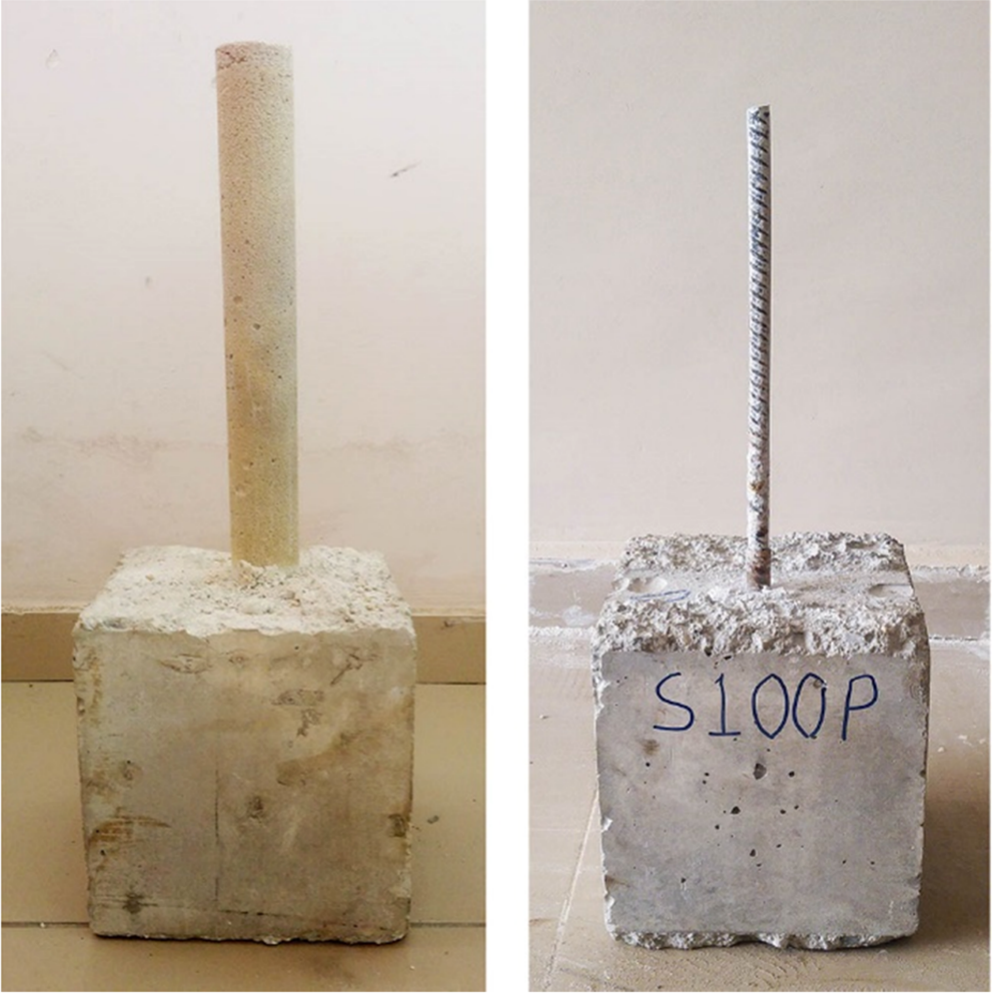
Pull-out sample coated with fire plaster and a sample ready for testing.

**Figure 4:**
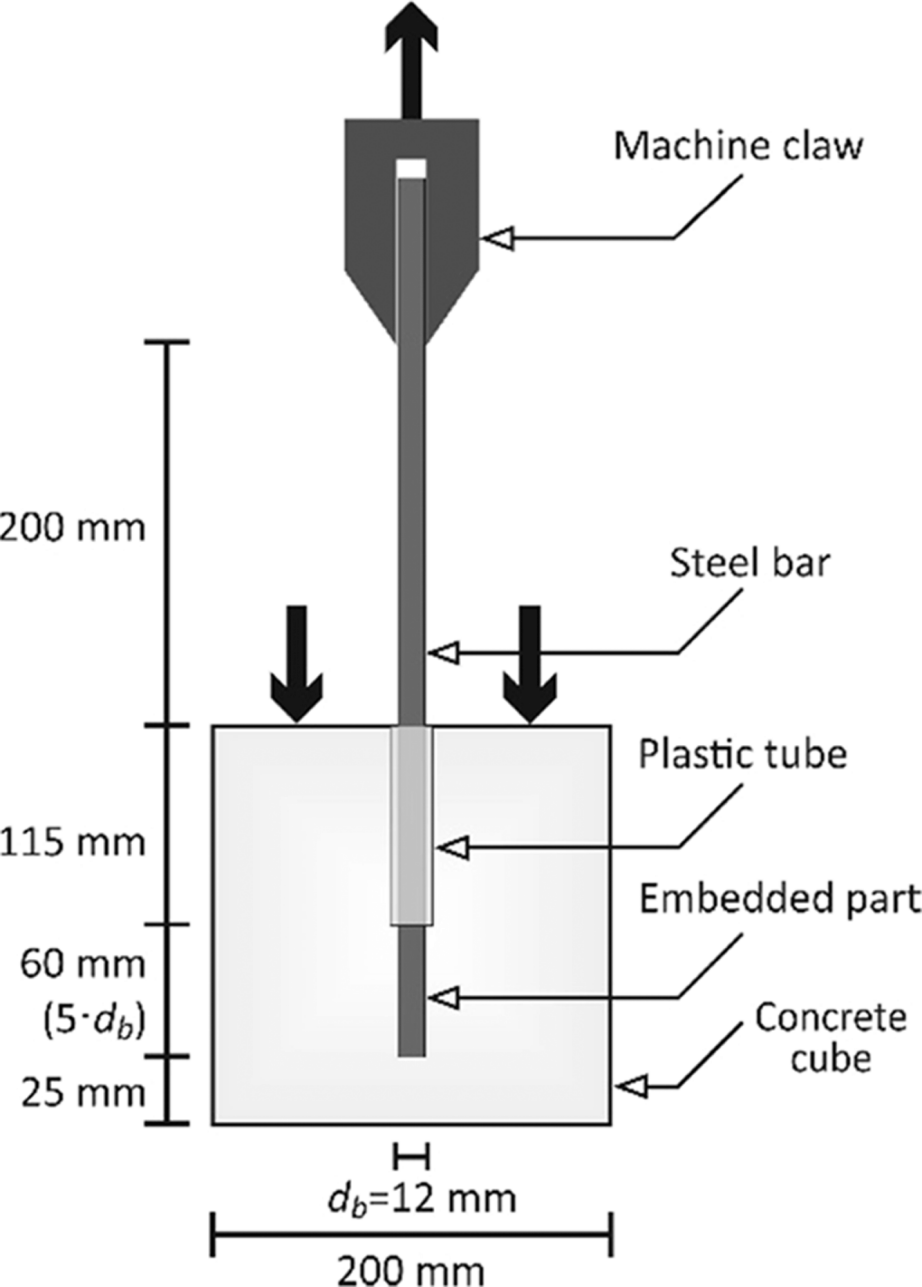
Pull-out test setup according to ASTM A944-10^32^ guidelines.

**Figure 5:**
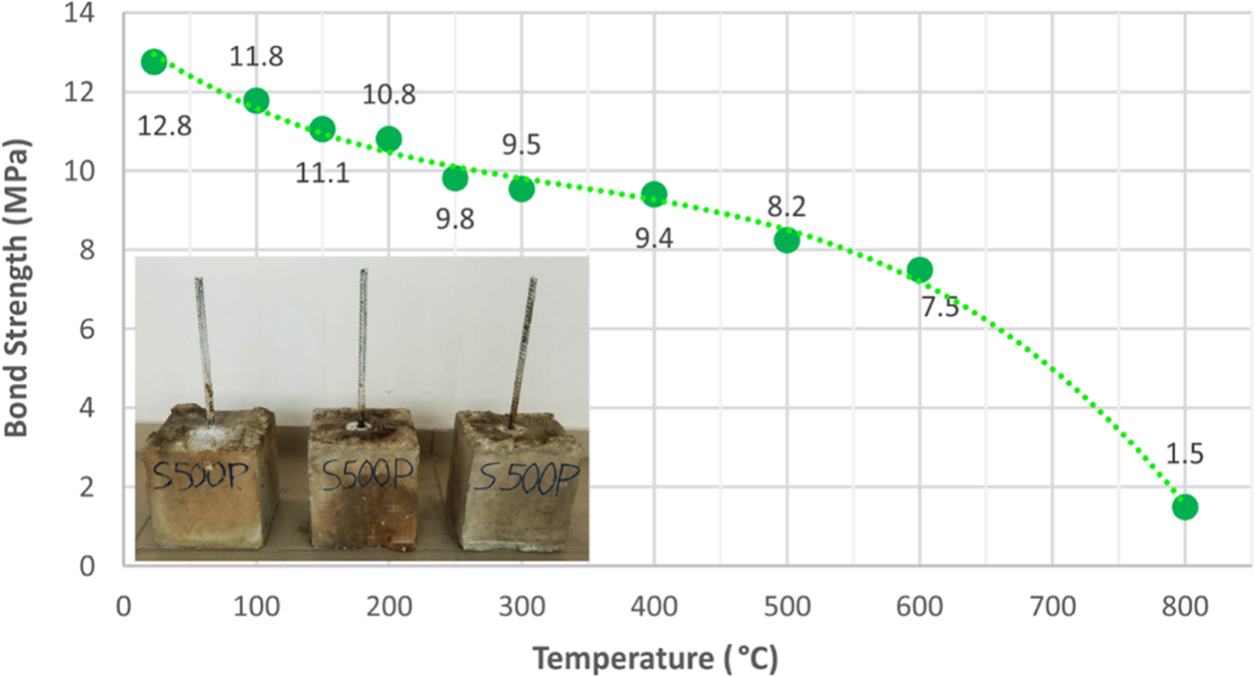
Bond strength of steel exposed to elevated temperatures.

Steel reinforcement has been subjected to pull-out tests according to ASTM A944-10^32^ guidelines. A displacement-controlled pull-out test procedure was performed for each of the rebars with a loading rate of 1 mm/min. Although related codes recommend testing with free-ends at the top and bottom faces of concrete cubes, this study aims to investigate the bond strength of rebars in order to simulate the real bonding behavior in RC members. Hence, steel bars were kept in the concrete by leaving a 25 mm cover at the bottom face of the concrete cube. The reinforcement is inserted into the concrete cube at a distance of five times the diameter, and the rest of the rebar is placed in such a way that it remains free in a plastic tube. In order to perform an accurate pull-out test, steel rebars embedded in concrete should be positioned axially. Therefore, steel rebars are fixed with the help of a stabilizer to ensure that they remain axially straight and do not change its position during concrete casting. The remaining part of the reinforcement outside the concrete, on the other hand, is protected with heat-insulating plaster (Figure 3) in order to prevent elevated temperatures to cause damage to the reinforcement outside the concrete and increasing the surface temperature between the reinforcement and the concrete sooner and by recognizing that the temperature is transferred to the reinforcement through concrete in a fire environment. The reason for the selection of the applied fire insulation plaster is that it is produced as a result of perlite expanded at approximately 900–1100 °C temperature levels, and it is known to be used in heat-tolerant brick materials as well.^31^ Rebar elongation results revealed that the steel material was not affected by the elevated temperatures. Hence, the 2 cm-thick applied fire plaster contributed to the accurate measurement of the test results. The pull-out test setup is schematically demonstrated in Figure 4. Before the test, the fire plaster (with T1 class thermal conductivity and A1 class fire resistance) on the outer part of the reinforcement was cleaned and subjected to testing. The bond strength curve of the steel corresponding to each temperature group is shown in Figure 5.

As the embedded parts of the rebars were not protruding from the bottom face of concrete cubes, axial displacement values were measured from the loaded end using internal transducers. The bond failure load of steel bars, being less than half of the yield strength, constrained the strain in the elastic region for steel pull-out specimens. Elongation of steel bars at each load level was calculated, accounting for the loaded-end length and the length in the plastic tube, to determine the slippage values by subtracting the bar elongation from the total displacement. Bond strength values for the rebars were determined based on surface stress, calculated as the ratio of the applied force to the embedded surface area of the rebars.

Considering the test results, it was found that about 70% of the bond strength of steel bars was preserved around 400 °C, the residual strength was about 60% at 600 °C, and almost 90% of the bond strength was lost at 800 °C.

For the purpose of fully comprehending the fire resistance and structural performance of reinforced concrete buildings, understanding the impact of elevated temperatures on the bond strength between concrete and reinforcements is of significant importance.^7,14,31^

## 3 Experimental Stage

### 3.1 Design of the Reinforced Concrete Beams

**Figure 6:**
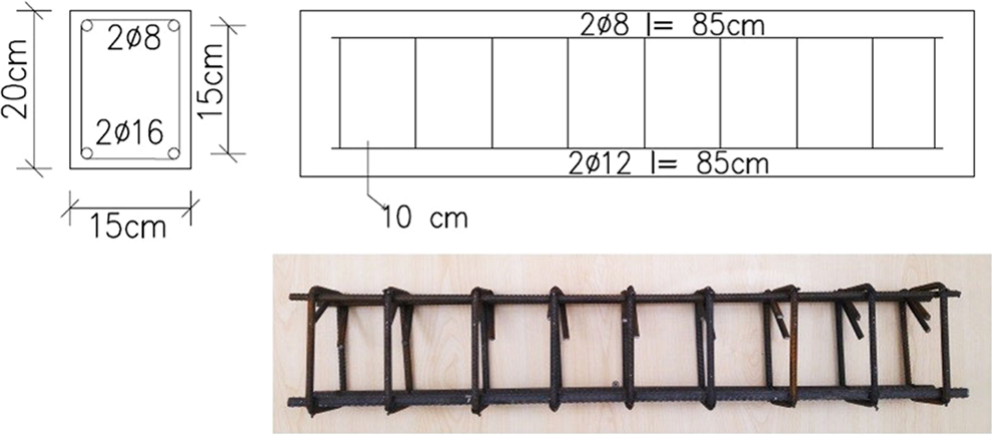
Reinforcement layout of the flexural failure group.

**Figure 7:**
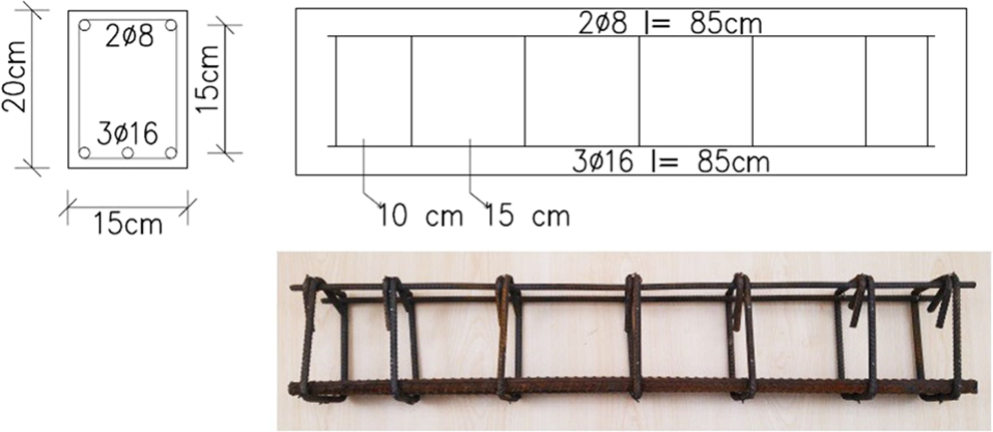
Reinforcement layout of the shear failure group.

Design of the reinforced concrete beams was performed by considering the results of the material tests. Two different designs have been made: flexural failure and shearing failure groups. For each group, ten reinforced concrete beams were produced to be investigated at different temperatures. The concrete cover for the test beams was adjusted to 2.5 cm in line with ACI 318-19.^33^ Design details of the beam specimens are given in Figures 6 and 7, and the design values are given in Table 1.

**Table 1:** Design Values of the Beam Specimens

	tensile reinforcement	tensile reinforcement ratio (%)	stirrup spacing (midregion)	stirrup spacing (end-regions)	shear capacity, *F*_u_^V^ (kN)	flexural capacity, *F*_u_^M^ (kN)
flexural failure group	2ϕ12	0.83	ϕ8/10	ϕ8/10	220	100
shear failure group	3ϕ16	2.20	ϕ8/15	ϕ8/10	170	240

### 3.2 Test Setup and Denotation

**Figure 8:**
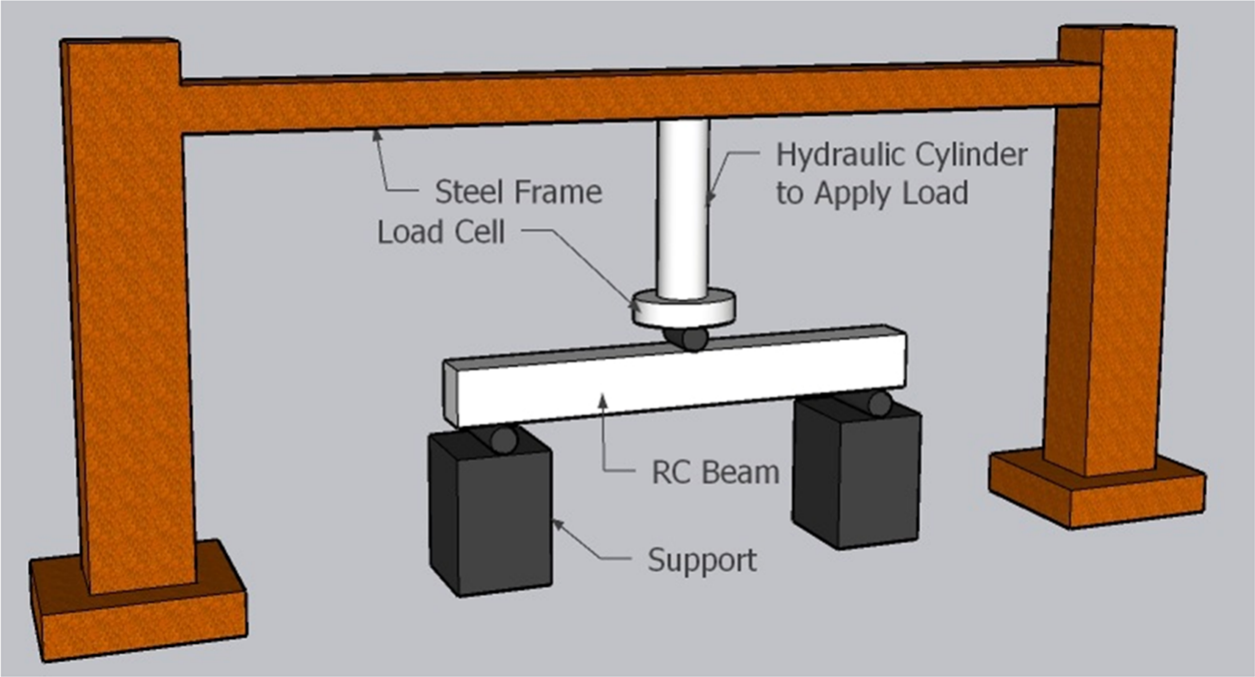
Test setup for the beam specimens.

The test setup was constructed with the help of a steel frame system, and the load was applied by gradually increasing on the beam specimens using a hydraulic cylinder having a push–pull capacity of 60 tons. To maintain a point loading, a steel cylinder was placed at a distance of 45 cm (at the exact midpoint) from the beam specimens. The test setup is demonstrated in Figure 8 within a detailed representation.

The test specimens were denominated considering that beams were reinforced with steel rebars as longitudinally and vertically, the degree of the exposure temperature, and the type of the failure group. For example, a flexural group specimen was exposed to 200 °C, and it will be called as SS200FL, or a shear group specimen will be called as SS200SH. Denotation for all of the specimens for both groups are given in Table 2. The specimens that were not subjected to elevated temperature were marked as “000” for the temperature identification since they were evaluated as control specimens despite the fact that they were tested actually at room temperature (23 °C).

**Table 2:** Denotation of the Beam Specimens

temperature (°C)	flexural failure group	shear failure group
23	SS000FL	SS000SH
100	SS100FL	SS100SH
150	SS150FL	SS150SH
200	SS200FL	SS200SH
250	SS250FL	SS250SH
300	SS300FL	SS300SH
400	SS400FL	SS400SH
500	SS500FL	SS500SH
600	SS600FL	SS600SH
800	SS800FL	SS800SH

After the beam specimens were casted in the molds and the setting of the concrete took place, they were placed in the curing pool for 28 days. The control specimens were tested at room temperature (23 °C) whereas others were exposed to the elevated temperatures and left to cooling. Considering previous studies, the ISO-834 standard fire effect was used as the heating regime.^14,15,18,19,31,34,35^ Then, for the purpose of examining the effects of heating on the specimens, they were subjected to the loading.

## 4 Experimental Results

**Figure 9:**
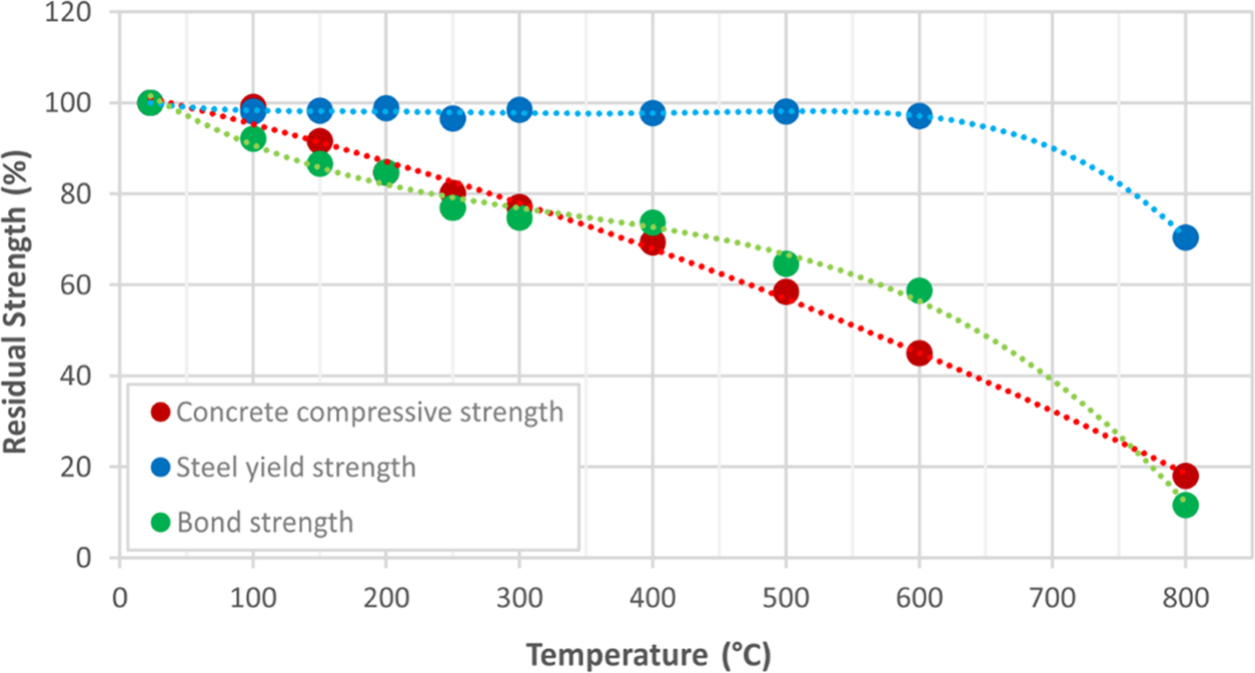
Residual strength values of concrete, steel, and bonding.

In order to express the results of the study more clearly, first, the preserved concrete compressive strength, steel tensile strength, and concrete-steel bond strength are given in Figure 9 in percentage.

As a result of the study conducted by Morley and Royles,^1^ it was found that there is a slight increment in concrete compressive strength up to 250 °C, but this situation reverses when temperature increases. However, in this study, a gradually accelerating downward tendency was observed in the compressive strength of concrete starting from 100 °C. According to the results, it could be highlighted that the main factor for the decrease in concrete compressive strength up to 250 °C is the heat exposure duration of the specimens in the furnace and advanced thermal expansion depending on this time. In other respects, as a result of a research conducted by Kadhum et al.,^13^ it was found that the protected concrete compressive strength was 67% at 400 °C, 58% at 600 °C, and 28% at 800 °C. Similarly, in this study, concrete samples were able to preserve 69% of the compressive strength at 400 °C, 45% at 600 °C and 18% at 800 °C.

Milke^26^ noted that typical structural steel crystallizes at 650 °C while Akman^4^ stated that the yield strength of steel falls below the safe zone at 600 °C. According to Lie^27^ and SFPE,^28^ steel preserves its tensile strength and stiffness by approximately 50% at 600 °C.^14^ Considering the tensile test results of steel rebars exposed to elevated temperatures in an unprotected state, there is no change for the steel yield strength up to 600 °C, while there is a loss of about 30% at 800 °C. Alonso et al.^5^ mentioned that yield strength will be regained after cooling at a maximum temperature of 600 °C for hot-treated steels, which is in parallel with the results of this study.

When the change of bond strength with temperature variation is evaluated, a gradual decrease is in sight. It was observed that 70% of the bond strength was preserved around 400 °C for steel rebars; this value decreased to 50% around 600 °C and almost all of the strength was lost. These results are similar to the outcomes of Bingöl.^36^ However, Bingöl^36^ diversely found that there was a slight increase in bonding performance up to 150 °C, which could be correlated to the different heating regimes of this study.

**Figure 10:**
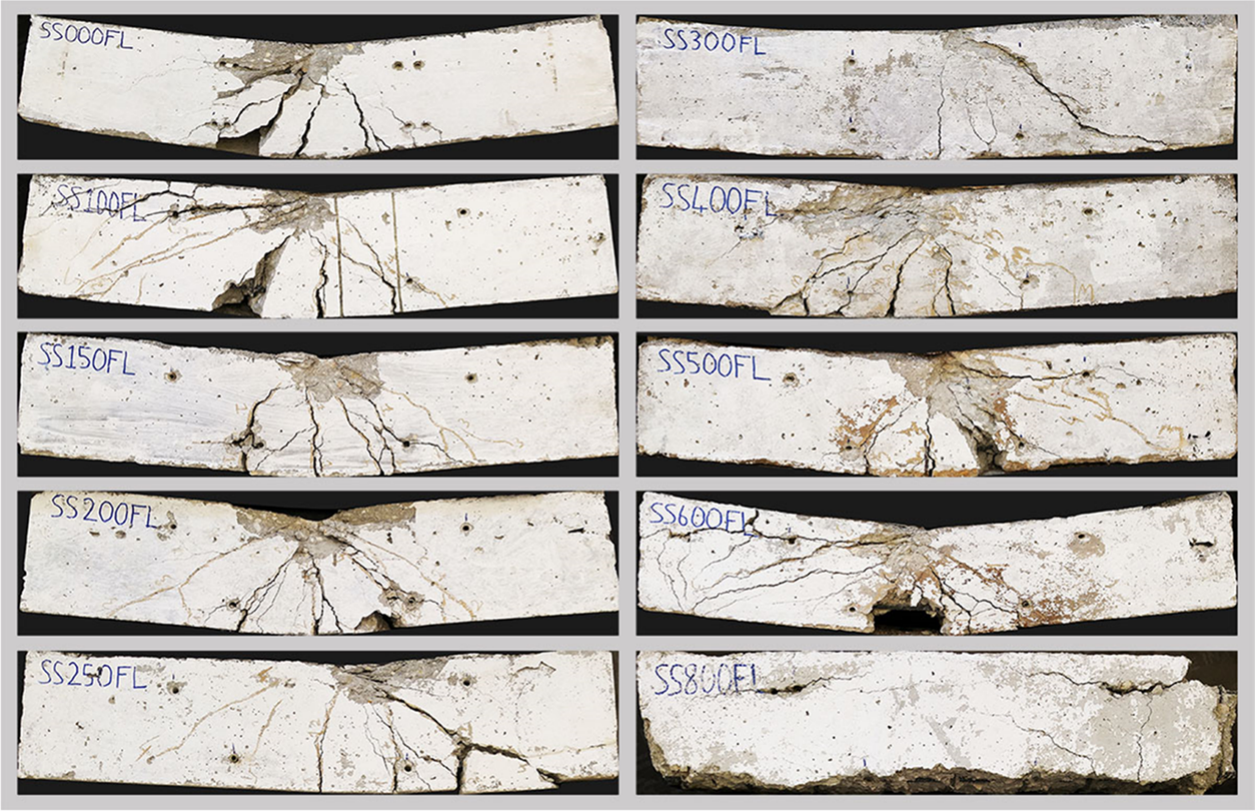
Flexural failure group after testing.

**Figure 11:**
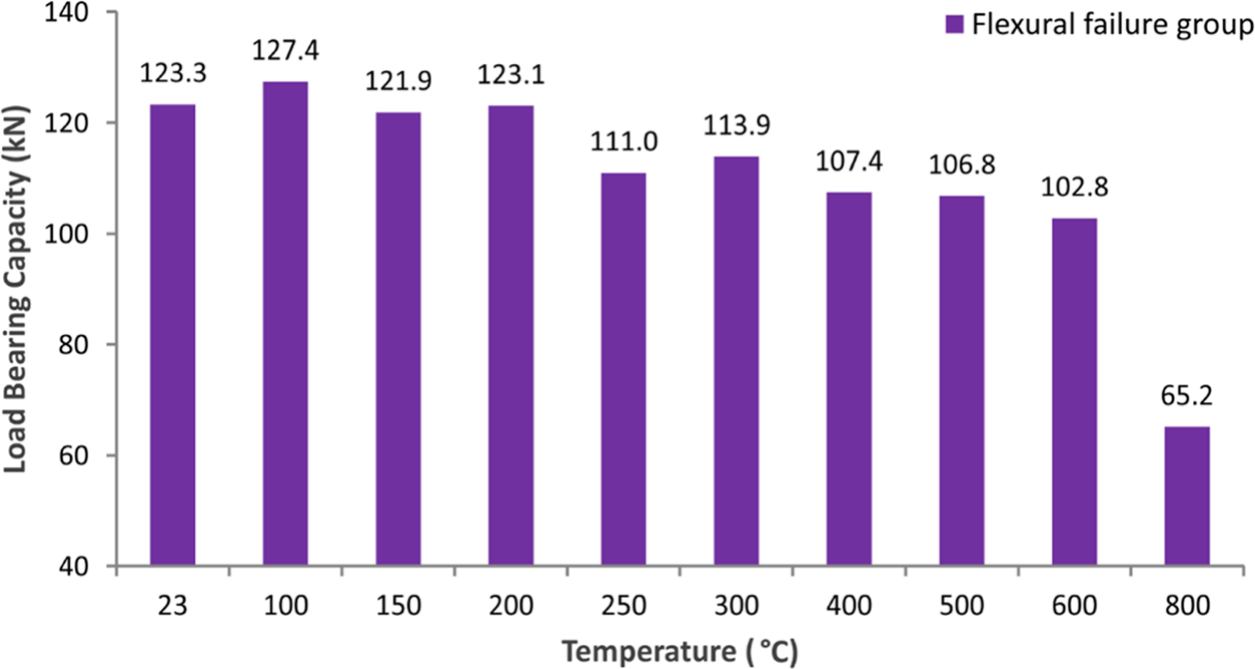
Load-bearing capacities of the flexural failure group.

Figure 10 shows crack formation in flexural group beam specimens. Cracks were formed in the flexural region of the beam and developed in a similar way to each other as expected. However, concrete crushing at the top level of the beam gets lost and bonding cracks become prominent as the temperature increases, especially after 600 °C. Finally, although the specimen seems to preserve its integrity, exposure to 800 °C caused the beam to lose half of the load-bearing capacity due to the deterioration of concrete and bond strength. Crack formation at 800 °C developed differently compared to other beams since the concrete completely loses its integrity, concrete cover is completely separated from the rebars and cannot provide any bonding even under very small loads at this temperature level. Figure 11 presents the load-bearing capacities of the beam specimens. Load-bearing capacities of the specimens up to 200 °C do not exhibit a significant change while capacity reduction is 10% at 250 °C, 14% at 500 °C, 17% at 600 °C, and 47% at 800 °C.

**Figure 12:**
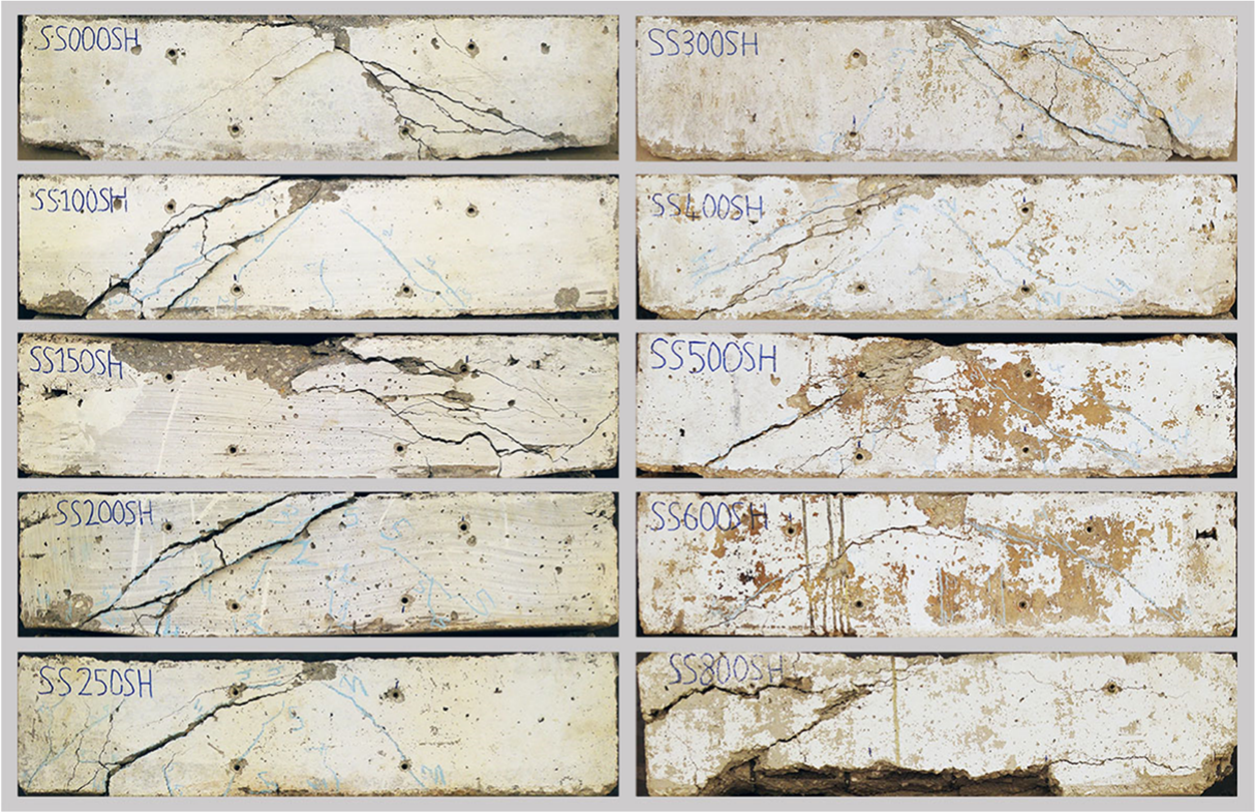
Shear failure group after testing.

**Figure 13:**
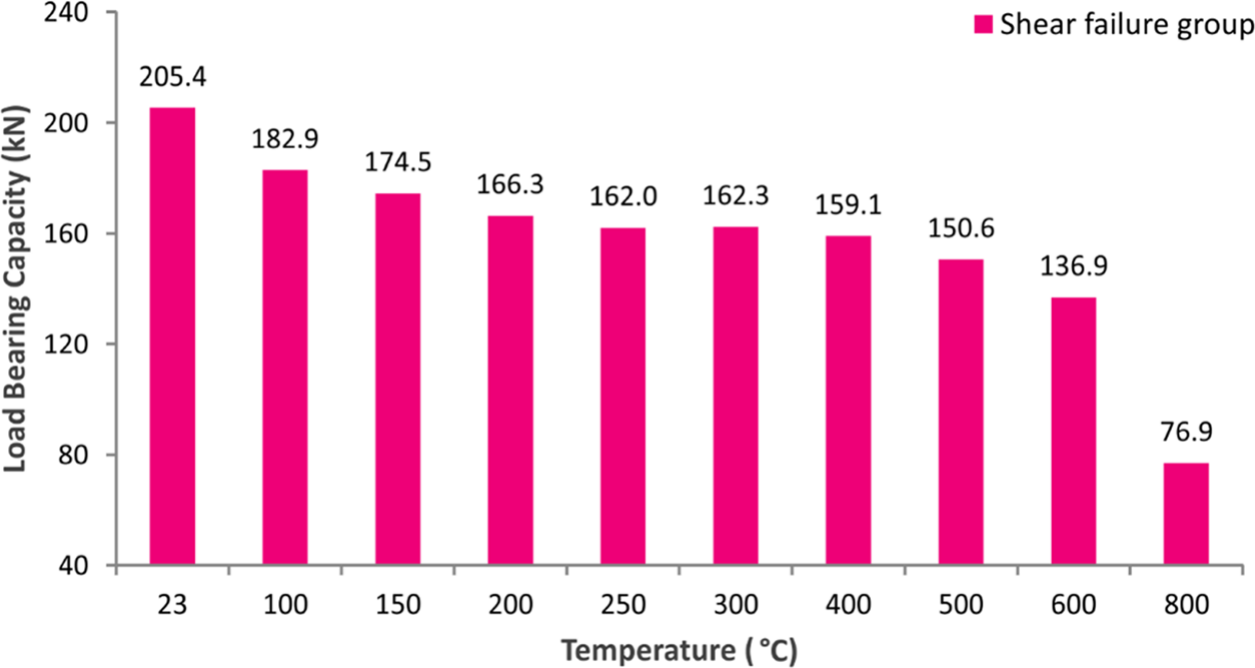
Load-bearing capacities of the shear failure group.

Figure 12 shows crack formation in shear group beam specimens. Regarding all of the specimens, cracks started to develop similarly to each other and ended in the support regions. However, crack formation at 800 °C appears to be different from the others same as the flexural failure group. Again, owing to the serious degradation of the concrete characteristics, the concrete cover layer falls from the bottom and the concrete cannot maintain its integrity. Figure 13 presents the load-bearing capacities of the shear group beam specimens, which decrease gradually after the control specimens. Capacity reduction is 21% at 250 °C, 27% at 500 °C, 33% at 600 °C, and 63% at 800 °C. It can be said that the main reason for the deformations and loss of load-bearing capacity under the influence of increasing temperature in all of the beam specimens is the deterioration of concrete, steel rebars, and bonding performance of especially stirrups.

**Figure 14:**
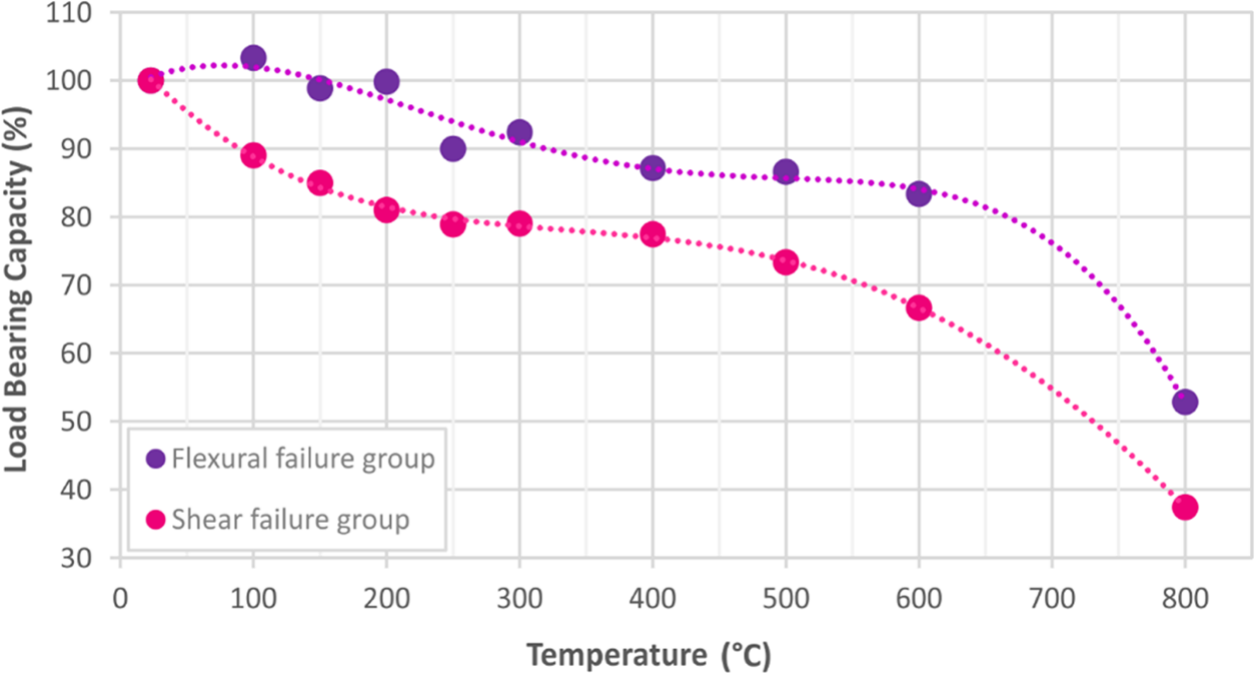
Load-bearing capacities of flexural and shear failure groups.

Load-bearing capacity results of the reinforced concrete beam specimens in shear and flexural failure groups (Figure 14) reveal that they exhibited a close behavior to each other. However, about 15% more strength loss was observed in the shear group compared to the flexural group. It can be said that the reason for this is that structural contribution of the concrete in the shear group is at the forefront, and concrete loses more strength under the effects of elevated temperatures than steel due to the thermal expansion parameter.

Within the experimental investigation, it was observed that there was no significant strength reduction in steel reinforcements up to 600 °C, and there was a 30% loss of strength at 800 °C. Considering concrete specimens, a 15% loss of strength was observed at 200 °C, reaching approximately 82% degradation at 800 °C. Based on these results for the beams under flexural effects, concrete crushes in the compression zone before principal steel rebars achieve yield strength. For the beams under shear effects, the loading ended with damage to the concrete block in the shear regions before the stirrups achieved yield strength. The main reason is the greater affection of the concrete under elevated temperature effects compared to the steel rebars, resulting in the loss of bond strength and the loss of the structural integrity.

## 5 Conclusions

Initially, material test results obtained from the study reveal that concrete compressive strength and steel yield strength values decrease at elevated temperatures within the expected tendency. As a reason for these results, expansion and contraction of the cement compounds and aggregates in concrete and deterioration of the molecular structure of steel rebars with respect to elevated temperatures are already known situations, and similar findings were reached in this study.

Although the downward trend of concrete compressive strength due to increasing temperature from 100 °C is consistent with previous publications, more tragic results were obtained. Heating duration and thermal expansion that occurred at a higher level depending on this time affected the mentioned decrease. While no significant degradation took place for the yield strength of steel rebars up to 600 °C, the appearance of strength losses after that level shows similarities with previous studies. It was also demonstrated with test results that the bond strength between concrete and steel reinforcement depends mainly on the compressive strength of concrete.

Considering the constancy of steel alongside the decrease of concrete compressive strength by 55% and the decrease of bond strength by 41% up to 600 °C, the main reason for the loss of load-bearing strength in both flexural and shear groups is the degradation of concrete compressive strength and concrete-steel bonding performance. This outcome gets stronger at 800 °C. Especially after 200 °C, shear cracks have also been observed to appear in the flexural group specimens due to the serious decrease in concrete strength. Again, for this reason, the loss of load-bearing capacity in the shear group specimens was more obvious compared to that of the flexural group. At 800 °C, it is essential to take precautions in reinforced concrete members against the effects of elevated temperature effects. Because the concrete loses its structural integrity, the bonding performance decreases to a notably low value and the reinforced concrete beams cannot exhibit an integrative behavior.

It has also been observed that crack formation in reinforced concrete beams becomes more serious due to increasing temperatures and the thermal expansion of the concrete. In addition, the deterioration of concrete and steel rebars in fere the fire-related temperature increment also degenerates the concrete-steel bonding and therefore directly affects the formation and development of cracks in reinforced concrete beams.
